# Role of poly-proline motif in HIV-2 Vpx expression

**DOI:** 10.3389/fmicb.2014.00024

**Published:** 2014-01-28

**Authors:** Ariko Miyake, Yasuyuki Miyazaki, Mikako Fujita, Masako Nomaguchi, Akio Adachi

**Affiliations:** ^1^Department of Microbiology, Institute of Health Biosciences, The University of Tokushima Graduate SchoolTokushima, Japan; ^2^School of Pharmacy, Research Institute for Drug Discovery, Kumamoto UniversityKumamoto, Japan

**Keywords:** HIV-2, SIV, Vpx, Vpr, PPM

Human and simian immunodeficiency viruses (HIV and SIVs) contain several auxiliary genes not found in other retroviruses. These genes are thought to be functionally important for optimal viral replication and persistence in infected individuals. Primate lentiviruses can be classified by the composition of these accessory genes. While viruses of the HIV type1 (HIV-1) group have *vif*, *vpr*, *vpu*, and *nef* genes, those of the HIV-2 group carry *vif*, *vpx*, *vpr*, and *nef* genes (Fujita et al., 2010). Vpx protein encoded by the *vpx* gene is unique to non-HIV-1 viruses, and is essential for viral replication in macrophages in contrast to its structural paralog Vpr (Fujita et al., 2010). The most outstanding sequence feature to distinguish Vpx from Vpr is the presence of poly-proline motif (PPM) at its C-terminal region. We have recently shown, by *in vitro* and *in vivo* assay systems, that the PPM in HIV-2 Vpx is critical for its efficient translation (Miyake et al., 2014).

**Figure 1:**
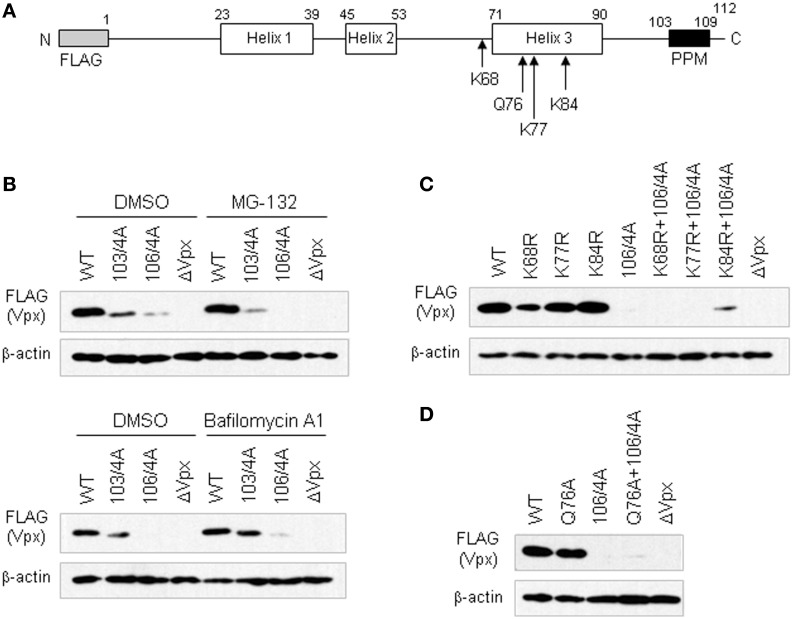
**Steady-state levels of various Vpx-PPM mutants in cells as monitored by Western blotting. (A)** Structure of the FLAG-tagged HIV-2 GL-AN Vpx construct. Numerals above the schema represent amino acid numbers of the Vpx protein. Positions of lysine and glutamine residues mutated are indicated. **(B)** Expression of Vpx-PPM mutants in the presence of a proteasome inhibitor MG-132 or a lysosome inhibitor Bafilomycin A1. **(C)** Expression of lysine-mutants with or without 106/4A mutation. **(D)** Expression of Q76A mutants with or without 106/4A mutation. For **(B)** to **(D)** experiments, 293T cells were transfected with the plasmids indicated, and harvested for Western blotting 24 h later. To examine lysosomal and proteasomal degradation processes **(B)**, 100 nM of BafilomycinA1 (Yoshimori et al., 1991) and 7.5 μM of MG-132 (McCulley and Ratner, 2012) were added at 5 and 16 h post-transfection, respectively. WT, pEF-Fvpx; ΔVpx, pEF-FxSt.

Although PPM consisting of seven consecutive prolines has been demonstrated to be required for efficient HIV-2 Vpx translation, thereby acquiring viral infectivity in macrophages, the effects of PPM mutations on the degradation of Vpx in cells was not formally analyzed as yet (Fujita et al., 2008; Miyake et al., 2014). Therefore, in this study, we asked whether the PPM plays a role in keeping away from proteasomal and/or lysosomal degradation (Figure 1). In order to assess this, we used various expression plasmids for HIV-2 Vpx (pEF-Fvpx series) described in a previous study (Miyake et al., 2014): wild-type (WT) plasmid has the *vpx* gene derived from HIV-2 GL-AN clone (Kawamura et al., 1994); mutants 103/4A and 106/4A have four consecutive alanine-substitutions at the site of P103-P106 and P106-P109, respectively, and have been shown to express a low/minimum level of mutant Vpx proteins in cells (Figure 1A); a negative control is a frame-shift mutant pEF-FxSt that lacks Vpx expression (ΔVpx).

Various expression plasmids were transfected into human 293T cells (Lebkowski et al., 1985) as described before (Adachi et al., 1986), and the amounts of WT and mutant Vpx proteins produced in cells in the absence or presence of a proteasome inhibitor MG-132 (Fujita et al., 2004; McCulley and Ratner, 2012) were comparatively examined by Western blotting (Miyake et al., 2014). A drastic reduction in Vpx expression was observed for mutants 103/4A and 106/4A, 106/4A in particular, both in the absence and presence of MG-132 (Figure 1B). These results showed that neither of these mutants could be rescued with MG-132, suggesting no involvement of the PPM in the proteasome-mediated degradation. Similarly, a lysosome inhibitor Bafilomycin A1 (Yoshimori et al., 1991) did not affect much the level of 103/4A and 106/4A in transfected 293T cells, although a small increase was observed for both mutants (Figure 1B). These results suggested that the low expression level of these PPM mutants may not be attributable to the lysosomal degradation.

Proteasomal degradation is generally triggered by the polyubiquitin modification of lysine residues in a protein. There are three lysines in the Vpx of HIV-2 GL-AN clone (Khamsri et al., 2006) (Figure 1A). We generated several clones carrying mutations in these residues. Furthermore, we focused on the 76th glutamine residue (Figure 1A). This amino acid has been reported to interact with DCAF1 for formation of Cullin4-based E3 ubiquitin ligase complex to degrade an anti-HIV restriction factor SAMHD1 (Hrecka et al., 2011; Laguette et al., 2011) by proteasome (Le Rouzic et al., 2007; Srivastava et al., 2008). Mutants K68R, K77R, K84R, and Q76A with or without the 106/4A mutation were constructed as described previously (Miyake et al., 2014) (Figure 1A), and examined for their expression in transfected cells (Figures 1C,D). As shown in Figure 1C, only one clone with K84R and 106/4A mutations showed a slight enhancement in agreement with a previous report (Srivastava et al., 2008). Moreover, no significant effect was observed for a mutant carrying Q76A and 106/4A mutations (Figure 1D). These results also suggested that PPM may not be associated with the proteasome-mediated degradation.

In total, proteasomal or lysosomal degradation does not account for the extremely low expression level of Vpx exhibited by the PPM mutants. This is consistent with our previous conclusion that PPM is critical for efficient translation of Vpx (Miyake et al., 2014). Molecular mechanism by which PPM enhances Vpx translation to a remarkable extent needs to be determined.
